# Abnormally low Bispectral index and severe hypoglycemia during maintenance of and recovery from general anesthesia in diabetic retinopathy surgery: two case reports

**DOI:** 10.1186/s12871-018-0510-z

**Published:** 2018-04-20

**Authors:** Chunhua Xi, Chuxiong Pan, Tianzuo Li

**Affiliations:** 1 Department of Anesthesiology, Beijing Tongren Hospital, Capital Medical University, Dongjiaominxiang 1, Dongchen District, Beijing, 100730 China; 2 Department of Anesthesiology, Beijing Shijitan Hospital, Capital Medical University, Tieyilu 10, Yangfangdian, Haidian District, Beijing, 100038 China

**Keywords:** Hypoglycemia, Bispectral index, Diabetes mellitus, General anesthesia

## Abstract

**Background:**

Hypoglycemia is one of the most fatal complications during the perioperative period. General anesthesia or sedation can mask a hypoglycemia-altered mental status. Acute hypoglycemia might result in permanent brain injury. There is no way to detect hypoglycemia during general anesthesia, except for intermittent blood glucose monitoring.

**Case presentation:**

Hypoglycemia is associated with changes in electroencephalogram readings. Here, we report two cases of patients with an abnormally low Bispectral Index (BIS) associated with diabetic retinopathy surgery, one in the recovery stage of general anesthesia and the other in the maintenance of general anesthesia. Hemodynamics were stable. Severe hypoglycemia (1.6 mmol/L and 2.2 mmol/L) was then detected. BIS increased with the correction of severe hypoglycemia.

**Conclusions:**

For diabetic patients, when the intraoperative BIS value is abnormally low, hypoglycemia should be considered. Severe hypoglycemia may be presented in BIS monitoring during general anesthesia.

## Background

The increasing prevalence of diabetic patients undergoing elective surgery has a significant impact on perioperative management. Many research studies emphasize perioperative hyperglycemia-related surgical mobility and mortality. However, perioperative hypoglycemia is found to be an independent risk factor for mortality [1, 2], and is also found to be associated with adverse outcomes after cardiac and non-cardiac surgery [3, 4]. Severe hypoglycemia is defined as an acute episode meeting two of the following criteria: 1) a blood glucose (BG) level less than 3.9 mmol/L or the presence of typical signs and symptoms of hypoglycemia in which active administration of carbohydrates or glucose is required, and 2) the need for external assistance, whether medical or non-medical [5]. Severe hypoglycemia can lead to cardiac ischemia, cardiac arrhythmias and myocardial infarction in the cardiovascular system [6, 7]. Severe hypoglycemia has also been associated with adverse neurological consequences, including cognitive dysfunction, coma and brain death [8, 9]. Therefore, severe hypoglycemia is one of the most fatal complications during the perioperative period.

According to the results of a self-report study, it is estimated that 5.2% of diabetic patients in China have had sight-threatening diabetic retinopathy (DR), and surgical intervention is necessary [10]. This number will rise with the increasing number of people with diabetes. Without intermittent BG monitoring, there is no way to detect hypoglycemia during general anesthesia or sedation during an operation. Here, we report two cases of severe hypoglycemia with an abnormally low Bispectral Index (BIS) during DR surgery. We suggest that we must be wary of the occurrence of hypoglycemia when the BIS value is abnormally low during general anesthesia for diabetes mellitus (DM) patients.

## Case presentation

A 56-year-old man weighing 53 kg was scheduled for silicone oil removal surgery due to silicone oil retention of the left eye. He had a 16-year medical history of Type 2 DM and had DR for 5 years. He underwent vitrectomy and silicone oil implantation of the left eye with local anesthesia nine months prior in our institute. His medicine included only the long-acting insulin analogue recombinant glargine, with 16 units prior to dinner every night by subcutaneous injection. His BG was well controlled, with the fasting blood glucose (FBG) at approximately 4–6 mmol/L, the postprandial blood glucose (PBG) at approximately 7–9 mmol/L and glycated hemoglobin (HbA1c) at 6.2%. He did not have any other medical history, and his laboratory tests including liver function and kidney function were normal. The operation was arranged for the next afternoon. The patient was asked to take the usual insulin dose the night before the operation and to fast until the time of the operation. At 6 a.m. on the morning of the operation, the FBG of this patient was 3.8 mmol/L. The patient received 500 mL of 5% dextrose with 5 units of insulin once, according to the order given by the ophthalmologist. The BG increased to 4.8 mmol/L at 8 a.m. The patient received another 500 mL of 5% dextrose with 5 units of insulin at noon, and his BG increased to 6.0 mmol/L at 13:00 before the surgery. General anesthesia was induced with atropine (0.5 mg), midazolam (1 mg), sufentanil (10 μg), etomidate (14 mg) and cisatracurium (10 mg) and maintained with propofol and reminfantanil. A laryngeal mask was inserted to support mechanical ventilation. A BIS was applied to monitor the depth of anesthesia. During the operation, the dosages of propofol and remenfantanil were titrated to 4–4.5 mg/kg/h and 0.03–0.04 μg/kg/min, respectively, almost half of the regular dose, and the BIS value was approximately 25–35. The hemodynamics of this patient were stable. To avoid intraoperative awareness, we did not decrease the dosage of propofol. The operation was successful, but the patient had no response to his name or prodding 15 min after anesthesia was stopped, and the BIS was approximately 35–40 without significant increase. Considering the low dose of anesthetics and his DM history, hypoglycemia was suspected. A finger stick blood glucose measurement showed that his BG was 1.6 mmol/L. His BG was then double-checked, and there was no error in the measurement. The initial intervention began with intravenous administration of 20 mL of 50% dextrose immediately and then was maintained with 500 mL of 5% dextrose. At 10 min after treatment, his BG was 4.6 mmol/L, and his BIS was approximately 50. At 20 min after treatment, his BG increased to 11.3 mmol/L, and his BIS was approximately 70–80. At 30 min after treatment, his BG was 10.7 mmol/L, and his BIS increased to 80–90. The patient could breathe then, but he could not move his limbs or legs. The laryngeal mask was pulled out, and the patient was transferred to the PACU. Approximately 15 min later, the patient could speak fluently and move his body according to instructions, and his BG was 10.0 mmol/L. This patient was sent back to the ward uneventfully, and he had no neurological injury during the next three days of follow-up.

The second case was a 41-year-old man with a 10-year history of Type 2 DM. He used Novomix 30 twice a day, and his BG was well controlled. He stopped Novomix 30 on the morning of the operation day. He also accepted 500 mL of 5% dextrose with 5 units of insulin twice, at breakfast time and at lunchtime. His BG was 4.8 mmol/L at 13:30 before the operation. General anesthesia was induced and maintained with the same drugs as the first patient. During the maintenance of general anesthesia, the anesthesiologist found that the BIS value was approximately 25–30, even if she reduced the dose of the anesthetics by half. The hemodynamics of this patient were also stable, and the capillary BG was checked. The finger stick BG was 2.2 mmol/L, and intravenous administration of 20 mL of 50% dextrose was started, followed by 5% dextrose. Approximately 15 min later, the BG increased to 5.1 mmol/L, and the BIS increased to 40–50. Approximately 30 min to an hour later, the BG was in the range of 6.0–7.8 mmol/L, and the BIS fluctuated steadily between 40 and 50. Recovery from general anesthesia was very smooth for this patient, and he also had no neurological injury during the next three days of follow-up.

## Discussion and conclusion

Hypoglycemia is the most common and severe side effect of insulin treatment for DM patients. Profound hypoglycemia can cause brain damage within a short time. The hippocampus, the amygdala and the frontal cerebral cortex are very vulnerable to glycemic control. Severely acute hypoglycemia might result in neuronal death and some cognitive defects [11]. Hypoglycemia-altered mental status can be observed when the patient is awake. Drowsiness can be seen when BG drops into the range of 1–2 mmol/L, and coma occurs when BG drops into a range less than 1 mmol/L [12]. General anesthesia or sedation masks these changes in mental status and might result in a severe brain injury [13].

The electroencephalogram (EEG) is a combination of brain electrical activity, which mainly reflects the excitatory and inhibitory postsynaptic activities in the cortex. Hypoglycemia is associated with changes in the EEG. At a median BG concentration of 2.0 mmol/L, the alpha activity (8–12 Hz) is decreased while the theta activity (4–8 Hz) is increased, and the alpha/theta ratio is sensitive to changes in the EEG during hypoglycemia in adults [14]. The EEG becomes flat when BG drops into the range of less than 1 mmol/L, and neuronal necrosis accelerates after 30 min of flat EEG in the rat model [12]. During the sleep stage, the EEG is full of low-frequency and high-amplitude slow waves, which is similar to that of general anesthesia. Hypoglycemia-induced EEG changes can be found in the sleep EEG pattern in adults; however, these changes cannot be distinguished from the sleep EEG of children [15].

Many studies have emphasized that hypoglycemia-induced EEG changes occur earlier than disorders of cognitive performance and neuroglycopenia, which makes it possible for a brain monitor to prevent brain injury from hypoglycemia [15, 16]. The EEG-based BIS monitoring has been recommended to guide anesthesia management, specifically to avoid intra-operative awareness and to improve clinical outcomes. The BIS value is calculated from three components: the spectral analysis, the bispectral analysis and the temporal analysis. The frequency domain-based relative β ratio is measured by the spectral analysis. The β ratio is defined as the logarithm of the power ratios in two empirically derived frequency bands: log(P_30-47Hz_)/(P_11-20Hz_), which predominate during sedation. The temporal analysis includes periods of flat EEG or nearly flat EEG, which can be seen under very deep anesthesia [17]. We found one case report describing an intraoperative insulin-related decrease in BIS and severe hypoglycemia (2.3 mmol/L) in a DM patient [18]. From the above discussion, we prudently suggest that the BIS may reflect severe hypoglycemia during maintenance of and recovery from general anesthesia to some extent. For DM patients, when the intraoperative BIS value is abnormally low and the overdose factor of anesthesia is excluded, BG should be measured immediately to avoid severe hypoglycemia and brain injury.

Finally, the two cases with hypoglycemia that we present here were given 5% dextrose with insulin and omitted two meals preoperatively. The purpose of preoperative fluid administration is to maintain intravascular volume, to provide energy and to avoid a disturbance of glucose metabolism for DM patients. However, our regimen is not suitable for every DM patient, especially for those patients with strictly controlled BG. The relative overdose of insulin, the fast infusion and the delayed action of insulin may cause hypoglycemia during the operation. Anesthesiologists should be aware of those people who have tightly controlled BG and accept insulin preoperatively. A consultation of multiple experts, including an endocrinologist, ophthalmologist and anesthesiologist, should be utilized to determine the preoperative fluid administration if the DM patient is omitting one or two meals before the operation.

In conclusion, general anesthesia masks hypoglycemia-altered mental status, which might result in severe brain damage and unacceptable sequelae. Many studies have described hypoglycemia-induced EEG changes during wakefulness and sleep. Here, we report two cases of patients who underwent diabetic retinopathy surgery and had an abnormally low BIS and severe hypoglycemia during the maintenance of and recovery from general anesthesia. We suggest that when the intraoperative BIS value is abnormally low for DM patients, hypoglycemia should be considered. Severe hypoglycemia may be reflected in BIS monitoring during general anesthesia.

## Abbreviations

BG: Blood glucose
BIS: Bispectral Index
DM: Diabetes mellitus
DR: Diabetic retinopathy
EEG: Electroencephalogram
